# Bio-Functional Design, Application and Trends in Metallic Biomaterials

**DOI:** 10.3390/ijms19010024

**Published:** 2017-12-22

**Authors:** Ke Yang, Changchun Zhou, Hongsong Fan, Yujiang Fan, Qing Jiang, Ping Song, Hongyuan Fan, Yu Chen, Xingdong Zhang

**Affiliations:** 1 School of Mechanical Engineering and Automation, Xihua University, Chengdu 610039, China; yangke493@163.com; 2 National Engineering Research Center for Biomaterials, Sichuan University, Chengdu 610064, China; hsfan@scu.edu.cn (H.F.); Fan_yujiang@scu.edu.cn (Y.F.); jiangq@scu.edu.cn (Q.J.); zhangxd@scu.edu.cn (X.Z.); 3 School of Manufacturing Science and Engineering, Sichuan University, Chengdu 610065, China; 2016223025082@stu.scu.edu.cn (P.S.); fanhy@scu.edu.cn (H.F.); 4 Department of Applied Mechanics, Sichuan University, Chengdu 610065, China

**Keywords:** metal implants, biomechanical design, porous structure, biodegradable metals, biological function design

## Abstract

Introduction of metals as biomaterials has been known for a long time. In the early development, sufficient strength and suitable mechanical properties were the main considerations for metal implants. With the development of new generations of biomaterials, the concepts of bioactive and biodegradable materials were proposed. Biological function design is very import for metal implants in biomedical applications. Three crucial design criteria are summarized for developing metal implants: (1) mechanical properties that mimic the host tissues; (2) sufficient bioactivities to form bio-bonding between implants and surrounding tissues; and (3) a degradation rate that matches tissue regeneration and biodegradability. This article reviews the development of metal implants and their applications in biomedical engineering. Development trends and future perspectives of metallic biomaterials are also discussed.

## 1. Introduction

After the invention of stainless steel in the 19th century, metal implants have been well-developed and widely used in biomedical applications [1,2,3,4]. During the 1960s and 1970s, a first generation of metal materials was developed as implants. A common feature of these implants was their biological “inertness”. Whereas, by the mid-1980s, bioactive materials had been proposed as second-generation biomaterials, in which biomaterials were designed to be either resorbable or bioactive [5,6]. Nowadays, more and more biofunctions are proposed for developing new generation of biomaterials which allow implants to interact with host tissues [7]. Regeneration properties become key feature for third-generation biomaterials, in which biomaterials are being designed to activate genes and cells to stimulate regeneration of living tissues [8]. The third-generation biomaterials combine multiple biological functions, with the aim of developing materials that, once implanted, will help the body heal or regenerate [9].

For metallic biomaterials, sufficient strength and inertness are two key features that should be considered in their early development [10]. Subsequently, the field of metallic biomaterials began to shift from inertness to bioactivity. This idea breaks old design principles for metallic biomaterials. More specific biofunctional designs in metallic biomaterials are proposed according to the application requirements [11,12]. Meanwhile, more design guidelines of metallic biomaterials need to be carefully considered. The first important issue is biomechanical design, including adequate mechanical properties such as strength, stiffness, and fatigue properties that are needed for appropriate design. Secondly, structure design and biological activation of metal implants are also crucial. Particularly, for bone tissue repair, surface bioactivation helps to integrate implants with host bones. Porous structure design is conducive to tissue growth and bone reconstruction. Thirdly, biodegradable design of metal implants is a new trend for regenerative tissue engineering. Biodegradable properties are important for novel metallic bone scaffolds or biodegradable stents. In many applications, implants are only needed temporarily and are expected to be biodegradable after supporting the healing process [13,14,15,16,17].

The biological functional design of metal implants is very important for clinical applications. Novel metal implants with different biological functions provide effective approaches for human tissue repair and regeneration. Innovative metal implant design and application are the most interesting issues in biomedical engineering. This paper reviewed three classic biological designs and applications of metal implants. It contains three main parts, the first part introduces the biomechanical design of metal implants, then porous structure design and biological activation of metal implants are introduced. Finally, as a new trend for regenerative engineering, the biodegradable design of metal implants is discussed. The state of the art of biodegradable metals, and their application for orthopedic and cardiovascular implants are reviewed. The future direction for metallic implants goes towards the combination of suitable mechanical property and bio-functionality. The study of innovative metallic implants is one of the most interesting research topics at the forefront of biomaterials.

## 2. Different Applications of Metal Implants in Clinic

**Figure 1:**
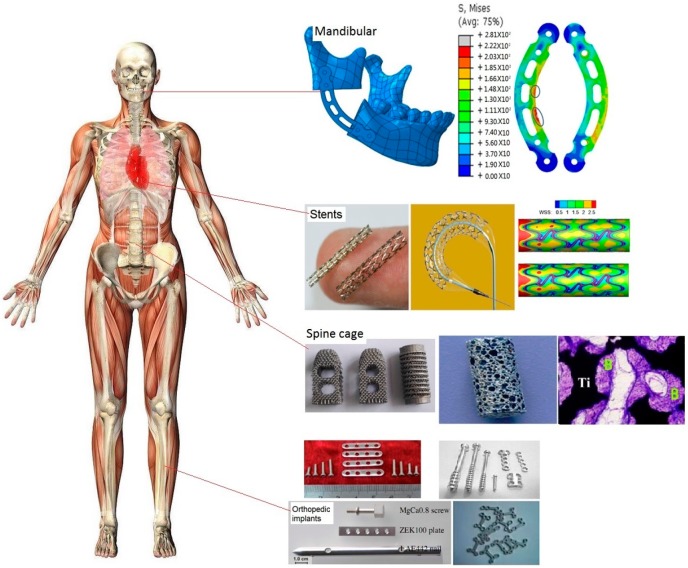
Different clinic applications for metal implants. Metal implants are mainly used in stents and hard tissue repair, which includes maxillofacial, spine and orthopedic fixation implants. WSS: wall shear stress; B: new bones.

Metal implants have been used for long time in the clinic. Metal implants are mainly used for hard tissue repair due to their excellent mechanical properties [18,19,20]. For example, titanium alloys are widely used in maxillofacial hard tissue repair. The complex stress state of maxillofacial bone tissues requires metal implants with matching mechanical properties to support mandibular functions [21,22]. Spine cages need metal implants with good osseointegration and high compressive strength to meet the needs of human movement. Hence, these implants were usually designed into porous structures to improve osseointegration and adjust mechanical strength [23,24]. For orthopedic implants, the biocompatibility and mechanical strength are critical concern issues. For stents which are hemolytic and bioabsorbable properties need to be carefully considered. Figure 1 shows several typical applications for metal implants.

### 2.1. Biomechanical Design of Metal Implants

The first important property of metal implants is the biomechanical design, including suitable mechanical properties such as strength, stiffness, wear, corrosion resistances and fatigue properties that need to be carefully designed [25,26,27,28,29]. A great number of researchers are still working on this field of biomechanical design to resolve various issues being faced today [30,31,32,33,34,35]. Biocompatibility and mechanical properties of metal implants are the most crucial properties for both temporary and permanent implants [36]. For metal implant mechanical design, biocorrosion plays an important role, especially when metal implants are used in load-bearing sites, such as screws for internal fixation of bone fractures [37,38,39]. In order to sustain pressurized loads, they must be stiff and able to resist deformation. Metal implants must also be light to facilitate motion [40,41,42].

**Figure 2:**
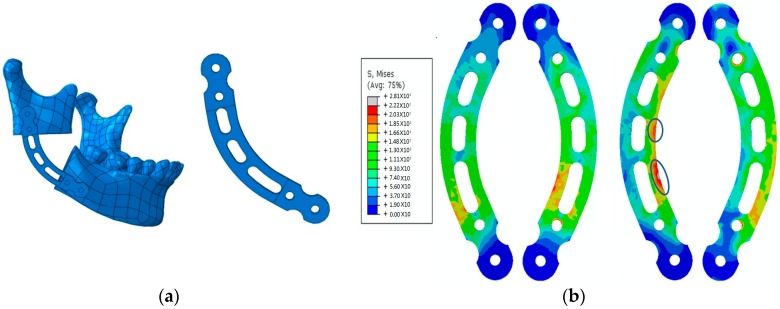
(**a**) **Left**: Models and grid; **Right**: a titanium reconstruction prosthesis. (**b**) The distribution of the strain of the von Mises on titanium prosthesis in different working conditions.

3D finite element modeling is a useful method which allows the analysis of the spatial stress of implants, and it has been widely used for the quantitative evaluation of stress spatial distribution on the implant. Tang et al. [43] proved the feasibility of topology optimization in the repair of mandibular defect and obtained a more mechanically suitable configuration of a titanium reconstruction plate, and provided suggestions for choosing and constructing the repairing configuration of mandibular defects in clinical treatment. The topology optimization on the configuration of a titanium reconstruction prosthesis is shown Figure 2.

Guo et al. [44] investigated stress and bone density distribution changes in the mandible due to the interference fit in titanium dental implants for mandible reconstruction, and studied the influence of interference magnitudes on mandibular bone remodeling.

**Figure 3:**
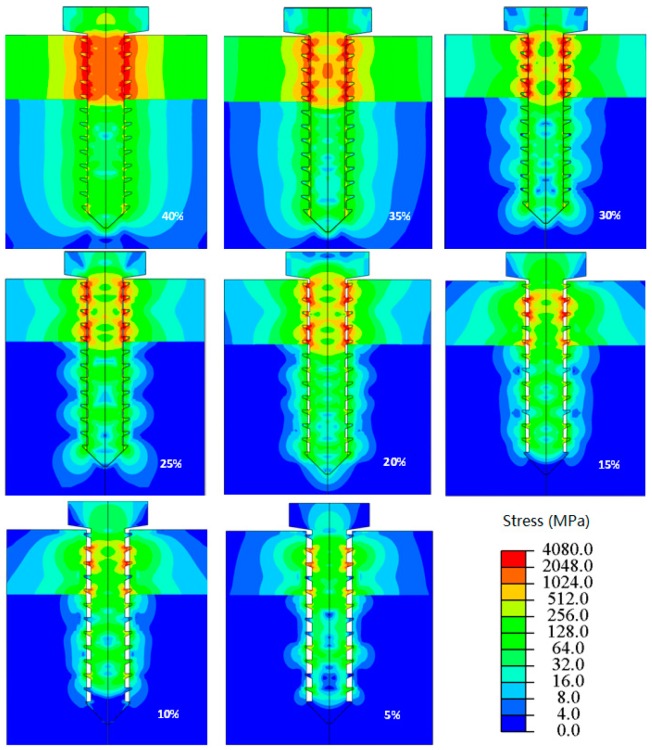
Von Mises stress distributions in models with different interference magnitudes after immediate implantation of titanium screw.

3D print metal technology has been developed in the field of medical bone implantation, Wan et al. [45] found stress concentration coefficients are significantly different for three distribution forms due to the difference of special location. Stress distribution of a single hole model is shown in Figure 3.

**Figure 4:**
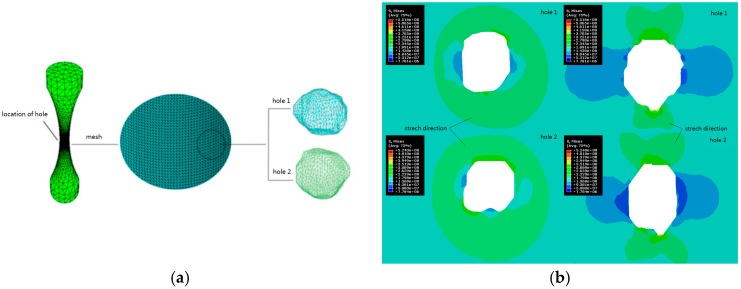
(**a**) Hole implantation and mesh generation; (**b**) Stress distribution of single hole model.

**Figure 5:**
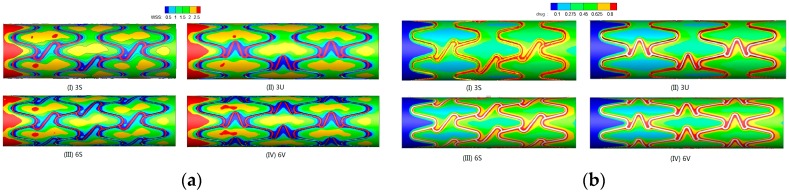
The distribution of WSS/drug concentration in different links DESs. (**a**) Wall shear stress (WSS), (I): Three S-type links, (II): Three U-type links, (III): Six S-type links, (IV): Six U-type links. (**b**) Drug concentration, (I): Three S-type links, (II): Three U-type links, (III): Six S-type links (IV): Six U-type links.

**Figure 6:**
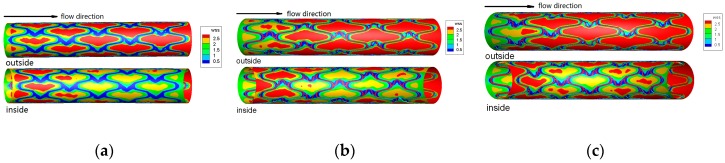
The distribution of WSS in different curvatures DESs. (**a**) 30°; (**b**) 60°; (**c**) 90°.

Stent implantation changed the intravascular hemodynamic environment, numerical methods were widely applied to the modeling of drug eluting stents and of their interaction with coronary arteries [46]. Chen et al. [47] compared different types of virtual drug-eluting stents (DESs) models with different links, geometries and curvatures, and analyzed the changes of hemodynamics and drug concentration caused by the implantation of three types of DESs with numerical simulation methods, including (1) the effects of DESs with different links on the drug concentration distribution; (2) the effects of DESs with different link numbers and geometries on the drug concentration distribution (as shown in Figure 4); (3) the effects of DESs with different curvature on the hemodynamics and drug concentration (as shown in Figure 5). Research on DESs provide valuable instructions and theoretical numerical conclusions that guide the design of DES (as shown in Figure 6) [47].

**Figure 7:**
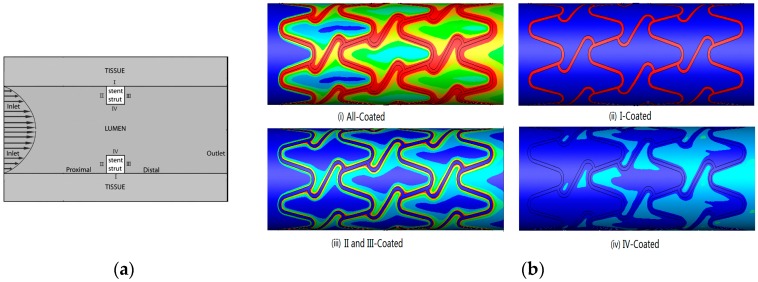
(**a**) Drug-coated Schematic diagram, the drug-eluting stent is nickel-titanium alloy and drug coated with rapamycin; (**b**) The drug distribution of four coated designs.

Changes of hemodynamics caused drug deposition and distribution on arterial walls, Chen et al. [48] analyzed four different coated models, and found drug coating on the contacting surface can provide effective drug release in the vascular wall without the interference from blood flow (as shown in Figure 7). It is possible to improve the uniformity of drug concentration in the vascular wall through optimal design of drug loading on the contacting surface.

### 2.2. Porous Structure Design and Biological Activation of Metal Implants

#### 2.2.1. Porous Structure Design and Manufacturing

**Figure 8:**
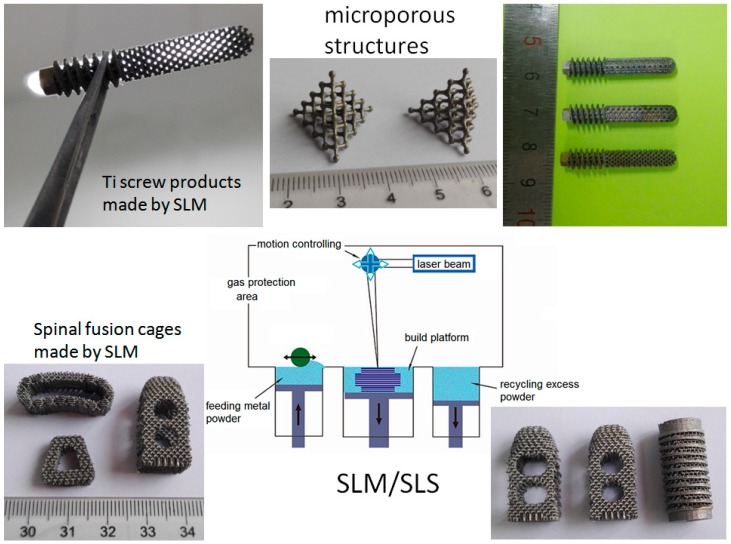
Some porous orthopedic implants fabricated by selective laser melting or selective laser sintering (SLM/SLS) technology. The upward arrow in SLM/SLS indicates that the platform of the printer is pushed up to provide print powders, and the downward arrows indicate the platform drop to recycle the print powders, bidirectional arrow means reciprocating pave the print powders.

For bone tissue substitutes, porous structure design and its manufacturing are very important for metal implants [49,50,51]. Porous architectures have effects on cell distribution and migration, as well as in vivo blood vessel formation, tissue ingrowth and sustaining new bone formation [52]. Recently, some advanced manufacturing technology have been proposed in biodegradable metal (BM) fields. For instance, by using 3D printing technology, the metals can be directly processed into scaffolds or implants [53,54,55,56]. In order to fabricate bone scaffolds, three-dimensional (3D) porous structures have been pursued to allow for bone ingrowth, to mimic the natural porous structure of bone. It has been possible to create a controllable porous, interconnected architecture via 3D printing technology. By using 3D printing, complex, customizable parts from metal powders can be directly manufactured into scaffolds with precise porosity [49,57,58,59,60]. Studies have indicated that the printed scaffolds demonstrated tensile mechanical property values very similar to those of natural bone, indicating its promise for bone replacement. Based on these preliminary studies, researchers believe that 3D printing will be a promising technology for manufacturing BM products [61,62,63]. Figure 8 shows some porous orthopedic implants fabricated by 3D printing technology.

#### 2.2.2. Biological Activation of Metal Implants

Biological activation of metal implants is another important issue for biomedical product applications [64,65,66,67] because every metal implant combined/integrated with host tissues through a bioactive interface or layer. It was found that certain biomaterials can bond to living bone through an apatite layer that forms on surfaces after being implanted [68]. As bioactive implants, the formation of bone-like apatite interface ability is an important factor for osteoinduction. It was well reported that a metal implant able to form the apatite on its surface in the living body is able to bond to living bone through the apatite layer, but an implant unable to form the surface apatite does not bond to host bones [69,70,71,72]. Therefore, many metal implant surface modifications were done to meet expected surface activation so that implants can bond to host bone tissues.

It has been proven that metal implants bond to living bone through an apatite layer that forms on their surfaces in the living body [73]. Since the 1980s, it has been reported that metals could be osteoinductive biomaterials through specific chemical and thermal surface treatments. Many studies reported that certain metal oxide gels, such as TiO_2_, ZrO_2_, Nb_2_O_5_, and Ta_2_O_5_ form the apatite on their surfaces in simulated body fluid (SBF) within a week. Metallic materials are generally covered with a thin oxide layer. These metals have bone-like apatite formation abilities on their surface, which means they have bioactivity [74]. Various surface modifications were applied to metal implants for inducing bone-bonding bioactivity, such as acid treatment, alkali or alkali-heat treatment, acid-alkali treatment, hydrogen peroxide treatment, anodic oxidation, electrochemical reaction and hydrothermal treatments. These methods are mainly based on chemical or electrochemical reactions occurring at the interface between the metal and surrounding solution. The mechanism of apatite formation can be explained as the electrostatic interaction between M–OH functional groups on the film and Ca^2+^, PO_4_^3−^ ions in the simulated body fluid between the metal and apatite [75,76,77].

**Figure 9:**
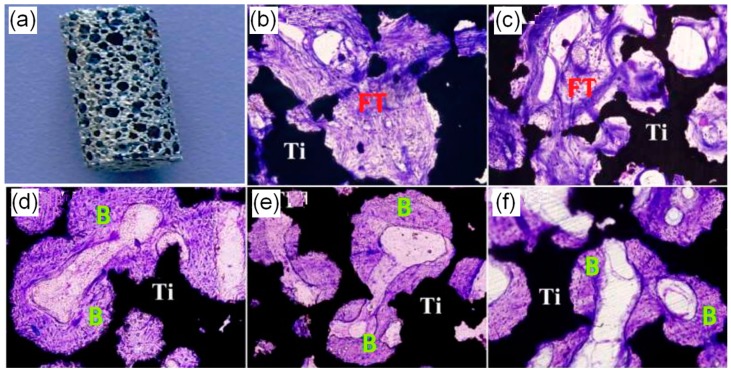
The osteoinduction phenomenon of in porous Ti metals. (**a**) is the porous Ti specimen, (**b**) is the histological observation after the Ti specimen subjected to NaOH treatment (NTPT), (**c**) is acid-alkali treatment (AAPT) specimen, (**d**) is hydrogen peroxide treatment (HOPT) specimen, (**e**) and (**f**) are hydrogen peroxide solution containing tantalum chloride treatment (HTPT) and chemical and thermal treatment (CTPT) specimens. Toluidine blue dye; FT: fiber texture; B: new bones; magnification: 200×.

Titanium and its alloys are the most widely used metal implants in orthopedic applications because of their good compatibility with living tissue [78]. Titanium was reported to show super apatite-forming ability, which allows them to directly bond to living bone in vivo [76,79]. However, in most cases, the bioactive properties only occur when appropriate surface treatments are conducted to modify and activate the metal surface. Surface treatments may change the surface microstructure and chemical composition of titanium. Porous titanium with different surface treatments showed excellent ability to induce bone-like apatite formation, and thus possessed in vitro bioactivity. For example, six types of surface treatments including NaOH treatment (NTPT), acid-alkali treatment (AAPT), hydrogen peroxide treatment (HOPT), hydrogen peroxide solution containing tantalum chloride treatment (HTPT) and chemical and thermal treatment (CTPT) performed on porous titanium showed different bone-like apatite-forming ability and in vivo osteoinductivity. By dorsal implanting for three and five months, ectopic bone formation was found histologically in most porous titanium scaffolds after implantation in the thighbone of adult dogs for two months (as shown in Figure 9). The osteoinduction phenomenon was found in porous Ti metals subjected to HOPT, HTPT and CTPT treatments. However, no obvious osteoinduction phenomenon was observed in NTPT and AAPT specimens. These results demonstrated that specific surface treatments could endow porous titanium with apatite-forming ability, and induce new bone formation [80].

### 2.3. Biodegradable Design for Metal Implants

Traditional metallic biomaterials require metals with sufficient strength and improved corrosion resistance in the body. However, with recent development in biomaterials, the new concept of biodegradable metals (BMs), rather than inert biomaterials, has been dramatically developed [81,82,83]. In these applications, metals need only to temporary support the healing process, and thereafter to be degraded in regenerative engineering [84,85].

#### Degradation Mechanism of Metal Implants

As a key property for biodegradable metals, biodegradation rate should be carefully considered. The corrosion mechanisms and their influencing factors of biodegradable metals have been widely studied [83,86]. Classic degradation of BMs is mainly explained as electrochemical corrosion in vitro. Corrosion reactions of metal implants involve the following anodic dissolution of the metal in physiological environment, the corresponding degradation reactions are given in Equations (1)–(4):

Oxidation reaction: M → M^n+^ + ne^−^ (1)

Reduction reaction: 2H_2_O + 2e^−^ → H_2_(g) + 2OH^−^ (2)

Reduction reaction: 2H_2_O + O_2_ + 4e^−^ → 4OH^−^ (3)

Product formation reaction: M^n+^ + nOH^−^ → M(OH)_n_ (4)

**Figure 10:**
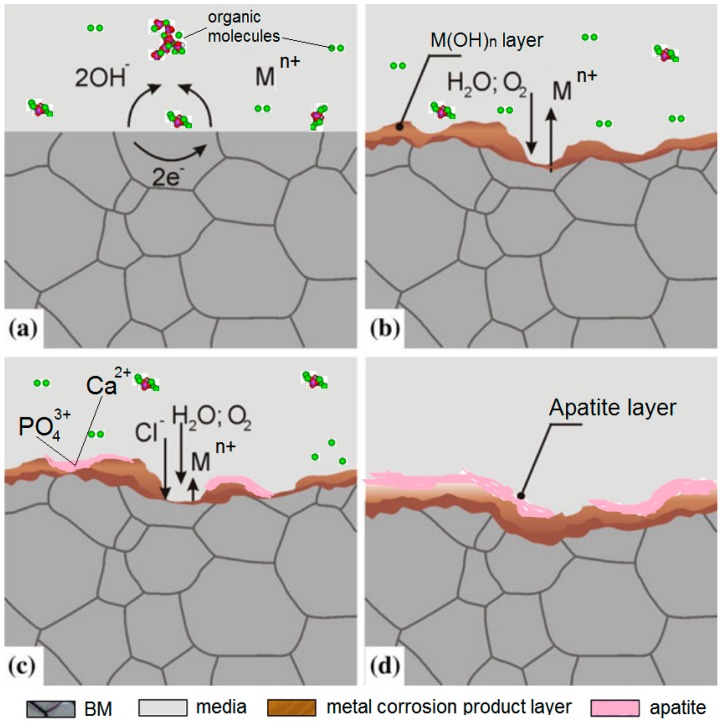
The explanation of degradation and apatite formation process on the surface of biodegradable metals (BMs). (**a**) is the metal implants just contact with body fluid, the oxidation-reduction reaction happened, the metals give away electrons formed anode, and the body fluid medium obtains electrons as cathelectrode; (**b**) is the corrosion happened and the metal corrosion product layer generated; (**c**) is the apatite layer formed and (**d**) is the final surface of the BMs.

The electrochemical corrosion reaction happen when the metal implants react with body fluid. In the oxidation reaction, the metals give away electrons and form positive ions. In reduction reactions, the body fluid medium obtains electrons as cathelectrode. From a chemical point of view, the biological environment is highly soluble for BMs, especially due to the presence of high concentrations of chloride ions in blood plasma. The biological environments have great effects on BM corrosion reactions. The corrosion mechanism and apatite formation process of BMs under human biological environments are shown as Figure 10. Ions present in the biological environment may strongly accelerate to the corrosion process.

## 3. Different Types of Biodegradable Metal Implants

Nowadays, different types of biodegradable metal implants have been well-developed and applied in clinical applications [13,87,88,89,90,91]. However, three main types of materials are mostly studied: (i) Mg-based BMs. (ii) Fe-based BMs. and (iii) Zn-based BMs. Among these BMs, Mg-based BMs are the most popular and have reported in many publications [92,93]. Fe-based BMs were reported recently in alloy design, and some animal testing was conducted for potential vascular stents. Zn-based BMs are less studied by researchers but seem to be promising candidates in the family of BMs. Table 1 shows some recent research progress of the three BM systems. Most research is focused on controlling the degradation rate, the in vitro cytotoxicity and animal testing.

### 3.1. Applications of Magnesium-Based BMs

Mg-based BMs have been successfully used because of their good biodegradable properties and biocompatibilities. Magnesium is an important element of the human body; an adult normally needs a daily intake of 300–400 mg magnesium. In blood plasma, Mg can be tolerated up to 85–121 mg/L [118]. Magnesium is also an essential element in bone tissue and it is beneficial to bone strength and growth. Promising Mg alloying systems including Mg–Zn, Mg–Ca, Mg–Sr, Mg–Si, Mg–RE, Mg–Mn and Mg–Ag have been well developed and investigated [84,119]. The manufacturing, microstructures, mechanical and biodegradable properties have been widely explored [120,121,122,123]. Magnesium alloys have excellent mechanical properties which have a large range of ultimate tensile strength (86.8–300 MPa) and elongation to failure (3–30%), respectively. Usually, Mg-based BMs degrade very quickly in the human body. The rapid degradation rate may cause the scaffolds to collapse shortly after implantation. Therefore, adding other alloying elements, microstructure adjustment and surface modification methods have been applied to control the biodegradation rate of Mg-based implants [81]. Addition of alloying elements, for example, adding Zn, Y, Ca, RE and Mn as alloying elements in WE43, ZW21 and WZ21, which may improve their corrosion resistance and generate exceptional plasticity of 17% and 20% [124]. These modified alloys exhibited fairly homogeneous slower degradation behavior in body fluids.

Some other methods such as mechanical deformation were used to improve the corrosion properties of Mg-based BMs. It has been reported that plastic deformation methods such as rolling, extrusion, drawing, forging and high pressure torsion can dramatically improve Mg alloys, mechanical properties and corrosion behaviors [13,125,126]. Surface modification and composition methods are also effective to control the biodegradation rate of Mg alloys. For example, AZ91D with hydroxyapatite (HA) coatings can slow down its corrosion process in SBF. AZ91 samples with coated polycaprolactone (PCL) layer can reduce their degradation rate.

### 3.2. Applications of Fe-Based BMs

Fe-based BMs are also good candidates for biodegradable stent applications because of their high strength (~1450 MPa) and high ductility (~80% elongation). High strength is favored for making Fe-based stents with smaller shape which is good for operation. High ductility is very important for expansion during the implantation of stents. However, Fe-based BMs show lower degradation rates [127]. Some attempts have been performed to accelerate the degradation rate of Fe-based BMs. For example, Mn, Pd, W, Sn, B, C, S and Si elements were introduced into alloys to accelerate their degradation rate. After adding Si into alloys, the corrosion rate of Fe_30_Mn_6_Si was higher than that of Fe_30_Mn alloy. Other alloying elements, such as Mn, Co, W, B, C, and S were found to have effects on improving the yield and ultimate strength properties, whereas the alloying element Sn led to a severe reduction in mechanical properties. Some studies on controlling of the manufacturing process to refine Fe-based BMs microstructures have been reported. Generally, the microstructure adjustment showed similar effects on modification of the corrosion rate.

Up to now, there is no clinical case report about Fe-based stents. Only several animal tests have been reported. Peuster et al. implanted Fe-BM stents into the descending aorta of New Zealand white rabbits and minipigs to examine their mechanical properties and biocompatibility [128]. The results demonstrated that the stent corrosion process is very slow and faster degradation rate is needed. Further studies focus on the modification of the composition and design of the stent to expedite the degradation process.

### 3.3. Applications of Zn-Based BMs

Zinc is another essential element for the human body. Normal adults contain 1.5–2.5g zinc, of which 60% exists in the muscle and 30% exists in the bone tissues. Zinc is also an important component of many enzymes; these enzymes help the synthesis of proteins and DNA, and promote cell regeneration and tissue metabolism [129,130]. According to the electrochemical principle, Zn has a moderate degradation rate which is faster than Fe but slower than Mg. Considering Mg-based BM degradation is too fast and Fe-based BMs degradation is too slow, Zn-based BMs are believed to be promising biodegradable metal candidates [131]. However, an obvious drawback of Zn-BMs is that pure Zn has very low strength and plasticity. In order to improve the mechanical properties of pure Zn, some elements were added into Zn-based BMs. It was found that Zn-BM Zn38Ca32Mg12Yb18 showed much higher strength (˃600 MPa) than conventional Mg based BMs (~200 MPa). This alloy also showed slower degradation rate than pure Mg and hardly any hydrogen is generated during implantation. Some studies have reported in vivo results on Zn-based BMs [131]. For example, pure zinc wires were placed into the arteries of rats and their degradation rate is about 0.2 mm per year, a perfect value for bioabsorbable stents in the first three months. However, after that, the corrosion accelerated, so the implant did not remain in the artery too long. Therefore, precise control of the degradation rate of Zn-based BMs will be a promising direction.

Along with the above three types of alloy systems, based on the principle that it is acceptable to add metal elements according to human body elements composition, Ca, K, Na, Si, Se, Cu, Mo, Sn, Co and Mn may be served as alloying elements. However, only a few new alloys containing these elements have been reported.

## 4. Stents Applications

Stenting, clinically known as percutaneous coronary intervention (PCI), has become a proven procedure for the treatment of coronary artery occlusions. During surgery, stents are delivered and placed into a narrowed coronary artery by using a catheter system that is inserted into the artery through a small incision in the arm or groin. Since its first use in biomedical applications in 1987 [132], stents have progressively been advanced from the conventional bare metal to the drug eluting and the most recent biodegradable stents [127,133].

Nowadays, biodegradable stents have been successfully applied in clinical trials [131,134]. Peeters et al. [135] reported that absorbable metal stent (AMS) (BIOTRONIK, Berlin, Germany) were implanted into 20 patients in 2005. The implanted stents were mostly degraded after six weeks. In 2005, Zartner et al. reported the first application of Mg-based stents [136]. In that case, a Lekton Magic AMS stent was successfully implanted into the pulmonary artery of a preterm baby. The degradation process had been completed after five months of implantation. In 2007, the BIOTRO–NIKGMBH and Co., Germany sponsored a series clinical trials to assess the efficacy and safety of AMSs in eight centers [137]. A total of 71 stents, 10–15 mm in length and 3–3.5 mm in diameter, were successfully implanted into 63 patients. That clinical trials showed good results with no myocardial infarction, subacute or late thrombosis or death of subjects during the study [138]. In 2013, Haude et al. [139] reported the first-in-man trial (BIO-SOLVE-1), which was conducted with 46 patients at five European centers. The 12-month results showed no cardiac death or stents thrombosis. As the above studies reported, the biodegradable stents had been optimized to provide much better degradation resistance than their predecessors with full degradation in 9–12 months. On 5 July 2016, the USA FDA announced the approval of Abbott’s Absorb GT1 bioabsorbable cardiac stenting system, which is the first fully bioabsorbable vascular stent in the world. However, only after one year, on 14 September, Abbott Laboratories announced that they will stop selling Absorb BVS in all countries [140]. Subsequently, Boston Scientific declared abandoning its biodegradable scaffold project [141]. Although the above fully degradable stents are polymeric based products, it alerts us that the design and evaluation of the degradability of metal stents are worthy of further study to avoid the associated potential risks when developing a new generation of BM stents.

## 5. Orthopedic Applications

**Figure 11:**
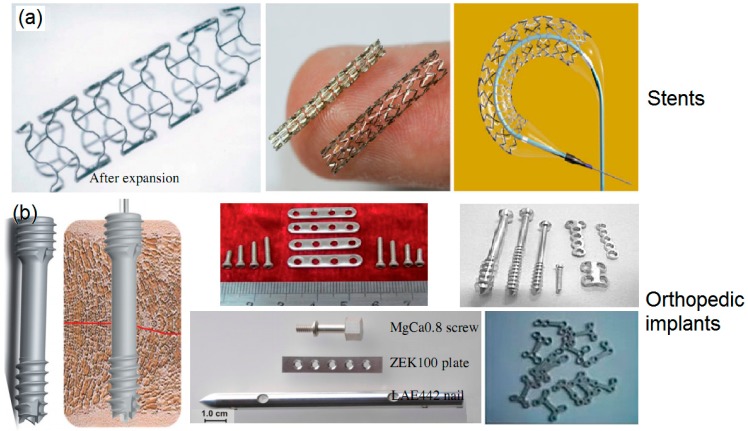
Two main application products: (**a**) stents and (**b**) orthopedic implants made of BMs.

Bones disease or bone fractures are very common in the clinic. Thus, fractured bone fixtures stimulate BMs to become a huge potential market in orthopedic applications [142]. Many Mg-based alloys such as ZEK 100, LAE442 and MgCa0.8 have been fabricated into screws for animal models and clinical trials [13,85,143,144]. A twelve months in vivo animal model confirmed that the Mg-based alloys showed positive osteogenetic effects after implantation. No gas generation was detected next to the implants of both. After 12 months of implantation of MgCa0.8 and LAE442 alloys, the implants showed osseointegration. Another in vivo study of a biodegradable MgYREZr-alloy screw in a rabbit model for 12 months revealed moderate bone formation with direct implant contact without a fibrous capsule. Histopathological results indicated this BM has good biocompatibility and osteoconductivity without acute, subacute, or chronic toxicity. Berglund et al. proposed a novel Mg−xCa−ySr system (x = 0.5–7.0 wt %; y = 0.5–3.5 wt %) for biodegradable orthopedic implant applications. In vitro cytotoxicity testing indicated that the Mg−1.0Ca−0.5Sr alloy is the most promising alloy for orthopedic implant applications since it showed lower degradation rate with no significant toxicity to MC3T3-E1 osteoblasts and a compressive strength of 274 ± 4 MPa [138]. Some other Mg-based system alloys also showed promising applications. MgCa0.8 screws showed comparable biomechanical properties as S316L screws in the first 2–3 weeks of implantation. The MgYREZr alloy showed equivalent strength to a standard titanium screw [139]. Figure 11 shows two main application products of BMs, i.e., stents and orthopedic implants [145].

## 6. Concluding Remarks and Perspectives

The biological functional design of metallic biomaterials is very important for clinical applications. The future direction for metallic implants goes toward the combination of suitable mechanical properties and multiple bio-functionalities. The study of innovative metallic implants is one of the most interesting research topics at the forefront of biomaterials. In comparison to the traditional bioinert metallic implants, BMs are representative bioactive biomaterials, and it should develop toward “third-generation biomaterials”. How to make sure the BMs play more active role in heal tissues, not simply as a tissue engineering scaffold, is the topic of continuous research. The interface between the bioactive implants and the host keeps a dynamic balance. The biodegradable rate of the implants, as well as the biodegradation products from implants, need a comprehensive consideration when designing novel metallic implants. The future trends and development direction of BMs are towards multifunctional capabilities. For example, novel metallic implants may provide temporary scaffolds with both structure size and mechanical strength requirements, with loading of drugs to prevent inflammatory reactions during implantation, and its biodegradation products/elements help local tissues reconstruction.

Benefiting from the development of some new manufacturing technologies, the metals can be directly fabricated into implants with multiple biological functions. For example, by using 3D printing technology, the implants can be printed into any porous structures with adjustable mechanical properties. It has been possible to create a controllable porous, interconnected architecture via 3D printing technology. By using select laser melting methods, a metal 3D printing technique, we can generate complex, customizable titanium implants from metal powders. Biomimetic porous structures have been designed to allow cell and fluid transportation and bony ingrowth. It is found that the 3DP method is capable of replicating highly accurate porous structure implants with errors in the range of 20 µm. Studies also demonstrated that the 3D printed implants show tensile mechanical properties similar to those of natural bone, and it presented an estimated corrosion rate because of 3DP has the ability to combine different materials in any space. Based on these developments, we believe that 3D printing is a promising technology for biomedical applications, and brings new opportunity for fabrication of novel metallic biomaterials with multiple biofuncitons.

The development of novel metal implants with different biological functions provides effective approaches for tissue repair and regeneration. There are three important design criteria for the new generation of metallic biomaterials: (1) mechanical properties with biomimetic design to those of host tissues; (2) porous structural design and surface bioactivation treatment; and (3) biodegradable metal design to match tissue regeneration. Third-generation metallic biomaterials are being designed to stimulate specific functions to meet diverse implant requirements, to perform as a drug delivery system, or to have cell and tissue specific properties. The separate design criteria of bioactive materials and resorbable materials need to converge. It is time to consider a shift toward multiple biological function design in metallic implants for the purpose of regeneration of natural tissues.

## Figures and Tables

**Table 1:** Research progress in Mg-based, Fe-based and Zn-based biodegradable metal implants.

Types of BMs	Materials	Published Time	Progress & Findings	Potential Applications
**Mg-based BMs**	ZEK100 [94]	2017	Biodegradable behavior of ZEK100 with various loading conditions were studied.	No declaration
	ZEK100 [95]	2016	Multilayered coatings carrying sodium alginate (ALG) were placed on ZEK100 to reduce the degradation rate.	Bone tissue repair
	Mg-Zn-Ca-Sr bulk metallic glasses (BMGs) [96]	2016	In vitro responses of bone-forming MC3T3-E1 pre-osteoblasts to Mg-Zn-Ca-Sr BMGs were studied.	No declaration
	Mg-3 wt % Zn alloy (MZ3) [97]	2016	Hot rolled Mg-3 wt % Zn alloy (MZ3) has been investigated for its potential in orthopaedic implant.	Orthopaedic implantations
	Mg-8Er-1Zn [98]	2015	A novel Mg-8Er-1Zn alloy with the ultimate tensile strength (318 MPa), tensile yield strength (207 MPa) and elongation (21%) were reported.	No declaration
	Mg-Zn-Ca-Sr alloy [99]	2015	Add minor Sr would improve glass-forming ability, mechanical properties, enhance and adjustable corrosion performance.	Orthopedic implantations
	Mg60Zn35Ca5 [100]	2015	Used first-principles molecular dynamics simulations to elucidate the structure of Mg60Zn35Ca5.	No declaration
	Nano-hydroxyapatite (nHA) reinforced AZ31 [101]	2014	Embedded nano-hydroxyapatite (nHA) particles enhance the biomineralization and control the degradation.	Skeletal implants
	AZ31 [102]	2014	Surface coating for Mg alloy AZ31 to control its corrosion rate.	Stents
	AZ31 [102]	2014	Report a new surface coating for Mg alloy AZ31 based on a low-toxicity ionic liquid, tributyl(methyl)phosphoniumdiphenylphosphate, to control its corrosion rate.	Stents
	RS66 [103]	2013	In vitro and in vivo experiments were conducted to analyze the biodegradation behavior and the biocompatibility.	Prosthesis implantation
	Mg-Zn [104]	2011	Biocompatibility test and biodagradation in vivo.	Orthopaedic implantations
**Fe-based BMs**	(Fe0.75B0.15Si0.1)100-xNbx (x = 0, 1 and 3 wt%) [105]	2016	Alloys exhibit excellent apatite-forming ability in simulated body fluids.	Stents and orthopedic implants
	Fe-based glassy alloys [106]	2016	It studied the multiple corrosion potentials in alkaline solution.	No declaration
	Fe-based metallic materials [107]	2015	Cytotoxicity of corrosion products of Fe-based stents relevant of pH and insoluble products were studied.	Stents
	Fe80-x-yCrxMoyP13C7 bulk metallic glasses [108]	2015	Alloys exhibit no cytotoxicity to NIH3T3 cells, and exhibit high corrosion resistance and excellent biocompatibility.	No declaration
	(Fe-10Mn-1Pd, Fe-21 Mn-0.7C-1Pd) [109]	2014	The study investigated the degradation performance of three Fe-based materials in a growing rat skeleton over 1 year.	No declaration
	Fe-Mn-C-Pd alloys [110]	2013	The research studied the alloying elements’ influence on metabolic processes.	No declaration
	Fe-Mn-Pd alloys [111]	2010	Fe-based alloys offering both an enhanced degradation rate and suitable strength and ductility.	Medical applications
	Fe(73.5)Si(13.5)B9Nb3Cu1 alloy [112]	2010	Studied the corrosion behaviors of amorphous and nanocrystalline Fe-based alloys in NaCl solution	No declaration
**Zn-based BMs**	Zn-Mg and two Zn-Al binary alloys [113]	2016	Alloys were fabricated by casting process and hot extrusion. Tube extrusion was applied to produce stents. Corrosion tests were performed.	Stents
	Zn-Mg alloy [114]	2015	Zn-Mg alloys with different Mg contents were prepared by melting-casting method. The Zn-3 wt % Mg alloy contributes to a general corrosion.	No declaration
	Zn alloys [115]	2013	Zinc exhibits ideal physiological corrosion behavior for bioabsorbable stents.	Stents
	CaZn based bulk glassy alloy [116]	2011	CaZn based glassy alloys shows low Young’s modulus, high fracture strength, good corrosion resistance and cytocompatibility.	Orthopaedic implantations
	Zn-Mg alloys containing 3 wt % Mg [117]	2011	The corrosion rates of the Zn-Mg alloys were determined to be significantly lower than those of Mg and AZ91HP alloys.	No declaration
